# Supplementary material for: Discovery and Transcriptional Profiling of Penicillium digitatum Genes That Could Promote Fungal Virulence during Citrus Fruit Infection
Source: J Fungi (Basel). 2024 Mar 22;10(4):235. doi: 10.3390/jof10040235 (PMC11051341; doi:10.3390/jof10040235)
Supplement: Supplementary file 1 [file jof-10-00235-s001.zip › tabla S1 oligos.pdf]

Table S1. List of oligos used in this study

Name	Sequence	Gene
**qVPdS3F**	TGCCTTCTCCCAATCAGCTG	**PDIP_28970**
**qVPdS3R**	AGGGAAGACCGGTTTGCAGTC	
**qVPdS7F**	ATGTCAACACTTGCACCAGCG	**PDIP_68700**
**qVPdS7R**	AGGGTTCTCGGTGCGAAGTCTG	
**qVPdS10F**	AGCTTACTCGCGCTCACTTC	**PDIP_38040**
**qVPdS10R**	ACTTCACCTGCAGATGGGAAG	
**qVPdS13F**	ATTGTGCACTCAGTCAGCAACG	**PDIP_64910**
**qVPdS13R**	TGCCATACTTCTGCTGCCATTC	
**qVPdS18F**	ATGACCTGCAGCGGATGCTC	**PDIP_41920**
**qVPdS18R**	TCCAACACGTTGTCGTAGGAAAG	
**qVPdS20F**	ATGATGCATTTGCAAGCCCGC	**PDIP_11000**
**qVPdS20R**	ATTCCTGTGCCTGGGAATATG	
**qVPdS21F**	ATCGCCAGGTGAACTAATAGGAC	**PDIP_00580**
**qVPdS21R**	ATCGCACCGTTGAACAGTAGC	
**qVPdS23F**	AGGATGTTTACGCCTGGTCCG	**PDIP_76190**
**qVPdS23R**	ATCGAGGGCTAGCTTGATAGAG	
**qVPdS25F**	AGGTGCAGAAACGGCTTGATC	**PDIP_05570**
**qVPdS25R**	ACCTAGCGCTCGATGAGTCG	
**qVPdS26F**	ACCGGCGCTCTTTGAAACTATC	**PDIP_01590**
**qVPdS26R**	AGCAAGACCTGTTGCTGGCG	
**qVPdS27F**	ATTCAGCGGGCATCTTCTCGAC	**PDIP_40660**
**qVPdS27R**	TCGGTGAGCATTTCCCGGATC	
**qVPdS34F**	ACATCACCGCGAGCACCAGTC	**PDIP_76160**
**qVPdS34R**	ACTTCGGTGACACGGCAATAG	
qVPdS48F	ACAATCGTGGCGCTGCTTCACC	**PDIP_66160**
qVPdS48R	AGGCCCTTGGCAGATCCCAT	
**qVPdS50F**	ACCTTCGAGCAGGCATCCGAG	**PDIP_49640**
**qVPdS50R**	ACCGTGGTTCTTGCCGAAG	
**qVPdS62F**	ACTTAGACAACATGTTCGGTCC	**PDIP_22490**
**qVPdS62R**	AGCCGATGAGTTAGAGTAAACAGG	
**qVPdS68F**	AGTGGTGCTATGGCTATCGCTC	**PDIP_74620**
**qVPdS68F**	ACCGCCGAGTCTACCACCGAG	
**qVPdS70F**	ACCGTGACTTGCTGCCTGAAC	**PDIP_78930**
**qVPdS70R**	ATGTCCACCGCGCTGGGAATATC	
**qVPdS74F**	AGCTATGCCAGCAGATGGGCTC	**PDIP_33600**
**qVPdS74R**	ACACACCCCCAGTTTTTGAAGC	
**qVPdS78F**	ATGGAGACCATGCAGTGGGGC	**PDIP_06990**
**qVPdS78R**	ATCAGTGACGACCCAGTCGAG	
**qVPdS84F**	ACGGCAACAACTCCAGTTCTG	**PDIP_49600**
**qVPdS84R**	GCCATAGCTAGAGGAGTCATTG	
**qTubF**	CGATGGCGATGGACAGTAAGTTT	**PDIP_27420**
**qTubR**	TTGGTTCGTGGTCGTTGTACTCA	
