# Supplementary material for: Discovery and Transcriptional Profiling of Penicillium digitatum Genes That Could Promote Fungal Virulence during Citrus Fruit Infection
Source: J Fungi (Basel). 2024 Mar 22;10(4):235. doi: 10.3390/jof10040235 (PMC11051341; doi:10.3390/jof10040235)
Supplement: Supplementary file 1 [file jof-10-00235-s001.zip › table S2 list genes.pdf]

Table S2. List of genes of VPdS cDNA substracted library. Those genes that were characterized with respect to their gene expression are shown in bold.

	Nº reads	Description
**PDIP_11000**	**12951**	**Hypothetical protein**
**Contig00054**	**11545**	**---NA---**
**PDIP_01820**	6901	CYP51-like; cytochrome P450 family 51
**PDIP_40660**	**6882**	**Glycerol uptake facilitator**
**PDIP_02280**	**4395**	**pectinesterase family protein**
**PDIP_49600**	**3737**	**Hypothetical protein**
**PDIP_66160**	**3109**	**Hypothetical protein**
**PDIP_33600**	**2676**	**C6 transcription factor**
**PDIP_05250**	1982	Nonsense-mediated mRNA decay protein Upf3
**PDIP_09350**	1914	40S ribosomal protein S4
**Contig00022**	1789	---NA---
**PDIP_49790**	1437	Hypothetical protein
**PDIP_26100**	1373	Hypothetical protein
**PDIP_28970**	**1252**	**Protein serine/threonine kinase (Ran1)**
**PDIP_68700**	**1233**	**Regulator of G protein signaling domain**
**PDIP_76160**	**1222**	**Camp independent regulatory protein**
**PDIP_05570**	**1096**	**Heat shock protein Hsp98/Hsp104/ClpA**
**PDIP_38040**	**1079**	**Cell division control protein cdc48**
**PDIP_22490**	**990**	**CorA family metal ion transporter**
**Contig00111**	837	---NA---
**PDIP_74560**	830	Mid2-like cell wall stress sensor
**PDIP_00960**	828	Ubiquitin carrier protein
**Contig00057**	821	---NA---
**PDIP_07120**	727	ToxD
**PDIP_71060**	632	Alkaline serine protease
**PDIP_76190**	**584**	**Hypothetical protein**
**contig00043**	581	---NA---
**PDIP_34230**	**566**	**Putative lysine-rich protein**
**PDIP_49590**	431	Hypothetical protein
**PDIP_00580**	**381**	**C6 transcription factor**
**PDIP_77110**	379	Helix-loop-helix DNA-binding
**PDIP_02870**	374	Hypothetical protein
**PDIP_15790**	351	60S ribosomal protein L13
**PDIP_64910**	**279**	**Hypothetical protein**
**PDIP_41920**	**231**	**Iron copper transporter**
**PDIP_75780**	222	Hypothetical protein
**PDIP_11260**	162	Hypothetical protein
**PDIP_01590**	**150**	**LYR family protein**
**PDIP_08950**	149	60S ribosomal protein L35Ae
**PDIP_54710**	147	40S ribosomal protein S13
**PDIP_30410**	145	60s ribosomal protein l6
**PDIP_15070**	126	60S ribosomal protein L7
**PDIP_01610**	124	Histone H2B
**PDIP_08960**	112	Glucose-6-phosphate 1-dehydrogenase
**PDIP_48610**	106	cyanovirin-n family protein
**PDIP_59000**	98	iron transport multicopper oxidase fet3
**PDIP_73430**	98	hypothetical protein PDIP_74560
**PDIP_61930**	92	Putative peroxiredoxin pmp20
**PDIP_25950**	91	Hypothetical protein
**PDIP_72240**	91	Cell wall beta-glucan synthesis
**PDIP_43290**	87	AhpC/TSA family protein

**PDIP_69680**	68	Mitochondrial import inner membrane translocase subunit tim14
**PDIP_07930**	50	Transmembrane protein UsgS
**contig00105**	48	---NA---
**PDIP_01290**	43	Helicase- DEXD box c2 type
**PDIP_39880**	42	Hypothetical protein
**PDIP_65180**	37	Hypothetical protein
**contig00076**	36	---NA---
**PDIP_18130**	36	MIPC synthase subunit (SurA), putative
**PDIP_09450**	35	40S ribosomal protein S9
**contig00094**	33	---NA---
**PDIP_80820**	33	Acyl-CoA N-acyltransferase
**PDIP_15610**	32	PaaI_thioesterase family protein
**PDIP_47340**	27	Fatty-acyl coenzyme A oxidase (Pox1)
**contig00093**	26	---NA---
**PDIP_21200**	26	RNA pol I specific transcription initiation factor Rrn7
**PDIP_74620**	**26**	**Mid2-like cell wall stress sensor**
**PDIP_83140**	26	Purine nucleoside permease
**contig00100**	25	---NA---
**PDIP_49640**	**24**	**Transcription factor (Snd1/p100)**
**contig00039**	22	Nucleic acid-binding, OB-fold
**PDIP_08150**	22	40S ribosomal protein S10a
**PDIP_21000**	21	Xyloglucan-specific endo-beta-1,4 -glucanase A
**PDIP_01300**	20	Hypothetical protein
**PDIP_21190**	20	Eukaryotic translation initiation factor eIF-5A
**PDIP_85230**	20	NEDD8-like protein (RubA)
**contig00114**	19	---NA---
**PDIP_69010**	17	Elongation factor 1-alpha
**PDIP_49530**	15	CipC-like antibiotic response protein, putative
**PDIP_78930**	**15**	**Calcium/calmodulin-dependent protein kinase**
**PDIP_03750**	13	DNA repair/transcription protein, putative
**PDIP_11440**	12	Mitochondrial F1F0-ATP synthase g subunit
**contig00096**	11	---NA---
**PDIP_18990**	11	60S ribosomal protein L28
**PDIP_13470**	10	Allergen Asp f 7
**PDIP_65450**	10	Importin beta-1 subunit
**contig00099**	9	---NA---
**contig00118**	9	---NA---
**contig00064**	8	---NA---
**PDIP_06990**	**8**	**Argininosuccinate lyase**
**PDIP_13100**	8	Hypothetical protein
**PDIP_16290**	8	60S ribosomal protein L38
**contig00108**	7	---NA---
**contig00080**	6	---NA---
**PDIP_45260**	6	CipC1, concanamycin induced protein C
**PDIP_65080**	6	Aspartate transaminase
**PDIP_73150**	6	40S ribosomal protein S12
**contig00103**	4	---NA---
**contig00123**	4	---NA---
